# Genome scaffolding and annotation for the pathogen vector *Ixodes ricinus* by ultra-long single molecule sequencing

**DOI:** 10.1186/s13071-017-2008-9

**Published:** 2017-02-08

**Authors:** Wibke J. Cramaro, Oliver E. Hunewald, Lesley Bell-Sakyi, Claude P. Muller

**Affiliations:** 1 Luxembourg Institute of Health, Esch-sur-Alzette, Luxembourg; 2 The Pirbright Institute, Ash Road, Pirbright, Woking, Surrey GU240NF UK

**Keywords:** *Ixodes ricinus*, Genome, Annotation, Haploid genome size estimation, Single molecule real time sequencing, Flow cytometry, Tick, Tick cell line

## Abstract

**Background:**

Global warming and other ecological changes have facilitated the expansion of *Ixodes ricinus* tick populations. *Ixodes ricinus* is the most important carrier of vector-borne pathogens in Europe, transmitting viruses, protozoa and bacteria, in particular *Borrelia burgdorferi* (*sensu lato*), the causative agent of Lyme borreliosis, the most prevalent vector-borne disease in humans in the Northern hemisphere. To faster control this disease vector, a better understanding of the *I. ricinus* tick is necessary. To facilitate such studies, we recently published the first reference genome of this highly prevalent pathogen vector. Here, we further extend these studies by scaffolding and annotating the first reference genome by using ultra-long sequencing reads from third generation single molecule sequencing. In addition, we present the first genome size estimation for *I. ricinus* ticks and the embryo-derived cell line IRE/CTVM19.

**Results:**

235,953 contigs were integrated into 204,904 scaffolds, extending the currently known genome lengths by more than 30% from 393 to 516 Mb and the N50 contig value by 87% from 1643 bp to a N50 scaffold value of 3067 bp. In addition, 25,263 sequences were annotated by comparison to the tick’s North American relative *Ixodes scapularis*. After (conserved) hypothetical proteins, zinc finger proteins, secreted proteins and P450 coding proteins were the most prevalent protein categories annotated. Interestingly, more than 50% of the amino acid sequences matching the homology threshold had 95–100% identity to the corresponding *I. scapularis* gene models. The sequence information was complemented by the first genome size estimation for this species. Flow cytometry-based genome size analysis revealed a haploid genome size of 2.65Gb for *I. ricinus* ticks and 3.80 Gb for the cell line.

**Conclusions:**

We present a first draft sequence map of the *I. ricinus* genome based on a PacBio-Illumina assembly. The *I. ricinus* genome was shown to be 26% (500 Mb) larger than the genome of its American relative *I. scapularis*. Based on the genome size of 2.65 Gb we estimated that we covered about 67% of the non-repetitive sequences. Genome annotation will facilitate screening for specific molecular pathways in *I. ricinus* cells and provides an overview of characteristics and functions.

**Electronic supplementary material:**

The online version of this article (doi:10.1186/s13071-017-2008-9) contains supplementary material, which is available to authorized users.

## Background

*Ixodes ricinus* is the most important European arthropod vector of human and animal pathogens and the most common tick species in Europe [1, 2]. It transmits a wide range of pathogens including bacteria, e.g. *Borrelia burgdorferi* (*sensu lato*), *Anaplasma* spp., *Rickettsia* spp.; viruses, e.g. tick-borne encephalitis virus; and protozoans, e.g. *Babesia* spp. The prevalence of ticks is on the rise throughout Europe due to environmental changes including climate [3, 4], forestry and wildlife management (e.g. increasing deer populations) [5–7]. Today, *I. ricinus* is found at higher latitudes and altitudes than just a few decades ago [8–10]. Moreover, longer questing activity periods have been reported [11]. In addition to deciduous and mixed forests and meadows, *I. ricinus* is also increasingly found in urban parks, recreational areas, private gardens and cemeteries [12, 13]. Epidemiological studies revealed that these urban tick populations are infected with tick-borne pathogens, such as *B.burgdorferi* (*s.l*.) (up to 18%), *Babesia* spp. (up to 3%), *Rickettsia* spp. (up to 8%), *Anaplasma* spp. (up to 16%) and *Ehrlichia* spp. (up to 16%) [14–18]. Therefore, *I. ricinus* ticks represent a considerable hazard not only for specific risk groups such as foresters, agricultural workers and livestock, but also for the general population and companion animals. To develop mitigation strategies against expanding tick populations and to reduce the risk of tick-borne infections, a better understanding of the *I. ricinus* tick and its genome is crucial. Genome coding sequences pave the way for comprehensive proteomic and transcriptomic studies. Publicly available reference genomes facilitate research in many ways including tick-host and tick-pathogen interactions and tick phylogenetics. Ticks may even be a resource for new pharmaceuticals such as e.g. anti-hemostatic agents [19] or complement inhibitors [20]. Homology analyses and genome annotation warrant functional analyses, potentially revealing new pharmaceutical targets for acaricide development and putative vaccine candidates. Despite the high prevalence of the *I. ricinus* tick in Europe and the scientific potential of a fully annotated genome, the first reference genome for this species was only recently published [21]. Here we further extend these studies by presenting a scaffold of the genome of the most important pathogen vector in Europe. The sequence information is complemented by the first genome size estimation for this tick species obtained by flow cytometry.

## Results

### Genome size estimation

**Fig. 1:**
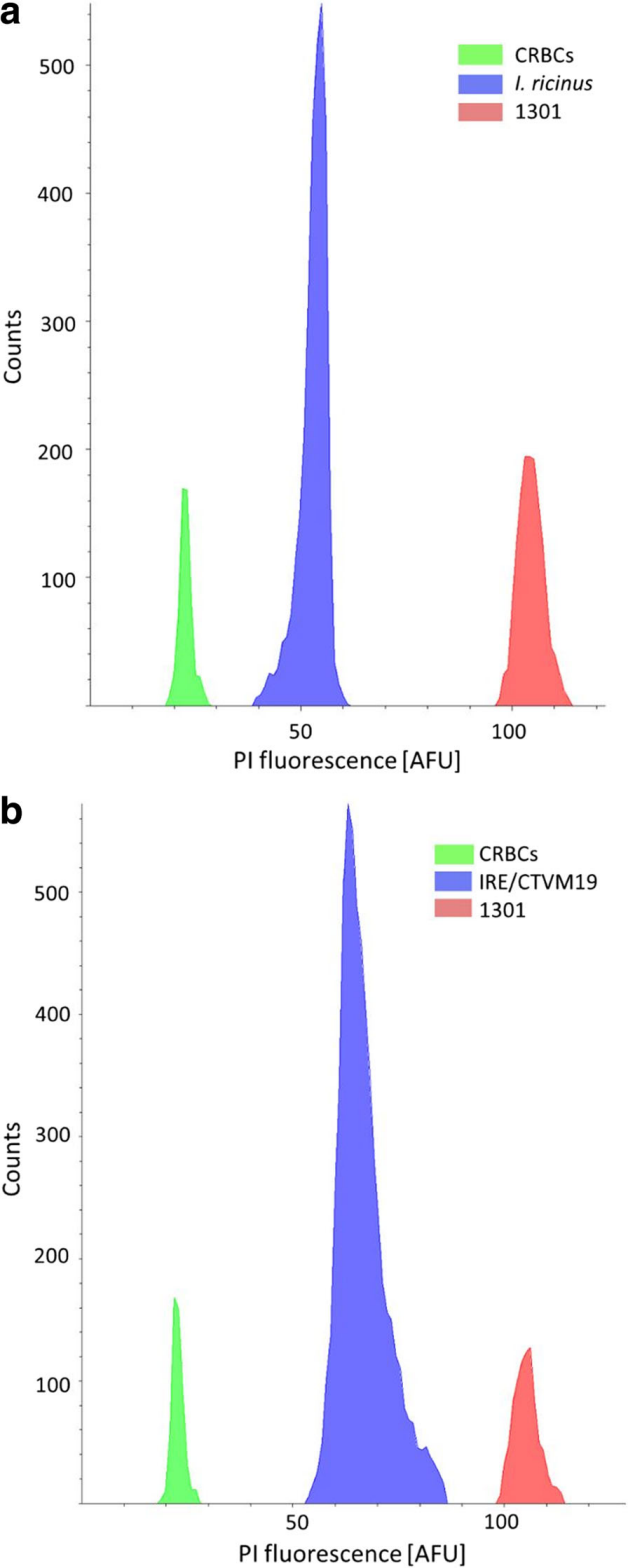
Genome size estimation for *I. ricinus* tick cells by flow cytometry. Cellular DNA was quantitatively stained with propidium iodide (PI). Chicken red blood cells (CRBCs) were stained with CFSE and 1301 tetraploid T cells (1301) with BV421 coupled CD45-antibody. Fluorescence was analyzed for *Ixodes ricinus* nuclei (*blue*; **a**) and IRE/CTVM19 cells (*blue*; **b**) as well as CRBCs (*green*; **a**, **b**) and 1301 cells (*red*; **a**, **b**). *Abbreviation*: AFU, artificial fluorescence units. Graphs are representative of 3 independent biological replicates per sample

The genome size of *I. ricinus* was estimated from tick cells of different origins including adult males and females of two laboratory colonies from Germany and Ireland as well as field ticks collected in Luxembourg. In addition, cells from the embryo-derived *I. ricinus* cell line IRE/CTVM19 were included. Cellular DNA was stained with propidium iodide (PI) and analyzed by flow cytometry. Chicken red blood cells (CRBCs) and the human tetraploid T cell line 1301 were used as internal standards (Fig. 1).

**Fig. 2:**
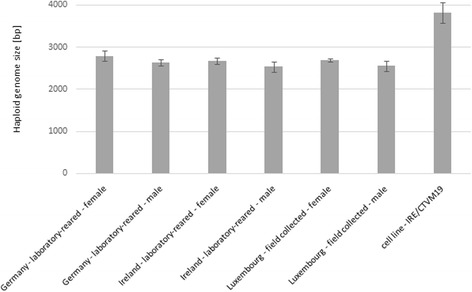
Genome size estimates for *Ixodes ricinus* tick cells from different origins by flow cytometry. The genome sizes of laboratory colony ticks from Insect Services (Germany) and Charles River (Ireland), field-collected ticks from Luxembourg as well as the *I. ricinus* embryo-derived cell line IRE/CTVM19 were analyzed by propidium iodide staining and flow cytometry. Error bars correspond to the standard deviation from 3 independent biological replicates

The genome size was calculated for each sample in direct comparison with the diploid CRBC standard and the tetraploid 1301 cell line standard and results were merged. The average size of the haploid genome of the *I. ricinus* strains analyzed was 2.72 Gb for females and 2.57 Gb for males. The overall average haploid genome size of both genders was 2.65 Gb. The genome size of the males was in all cases approximately 95% of the genome size of their female counterparts. Among the different strains analyzed, the laboratory colony from Germany had the largest genomes with 2.79 Gb for females and 2.64 Gb for males, while the laboratory colony from Ireland (2.67 Gb for females, 2.53 Gb for males) had the smallest genomes. The genome sizes of the ticks collected in Luxembourg were intermediate (2.69 Gb for females, 2.55 Gb for males). The genome of the IRE/CTVM19 cell line was, at 3.80 Gb, about 1.4 fold larger than the size of the average tick genome (Fig. 2). The genomes of the various ticks were not significantly different from each other, but the genome of the IRE/CTVM19 cells line was significantly larger (*P* < 0.001).

### Genome scaffolding

Genomic DNA from male *I. ricinus* ticks was sequenced with the PacBio RS system. This third generation SMRT sequencing technique generated 5,624,511 ultra-long genomic reads containing 11,843,540,746 bp. This corresponds to a 4.5-fold sequencing coverage of the *I. ricinus* genome with an average size of 2.65 Gb as described above. These sequences were combined with the sequences from the first *I. ricinus* reference genome based on an Illumina shotgun sequencing approach and described recently by our group [21].

The PacBio reads were mapped against the assembled contigs as a reference. PacBio reads spanning several contigs as well as reads spanning gaps or aligning into gaps, were identified and further analyzed. Thus, 5,584,233 PacBio reads (99%) were mapped to extend and scaffold the reference contigs and fill the gaps. As a result, 235,953 contigs were integrated into 204,904 scaffolds. The total genome lengths spanned was extended from 393 to 516 Mb, which represents 19.4% of the *I. ricinus* 2.65 Gbp haploid genome*.* A comparison of the new scaffolds with the original reference contigs is shown in Table 1.

**Table 1:** Comparison of the created scaffolds to the original reference contigs

	Contigs	Scaffolds
Number of sequences	235,953	204,904
N50 (bp)	1643	3067
Longest sequence (bp)	32,538	38,109
Total lengths spanned (bp)	392,924,918	515,788,051

A gap was defined as a stretch of at least 25 unknown nucleotides. Six hundred and forty-eightof the 204,904 scaffolds (0.3%) contain gaps resulting from low quality base calling in these positions. The longest gap is 141 bp in length, the mean gap length is 57 bp. In total, 37,176 nucleotides of the 515,788,501 bp (0.007%) are unknown.

This Whole Genome Shotgun project has been deposited at DDBJ/ENA/GenBank under the accession JXMZ00000000. The version described in this paper is version JXMZ02000000.

### Genome annotation

**Fig. 3:**
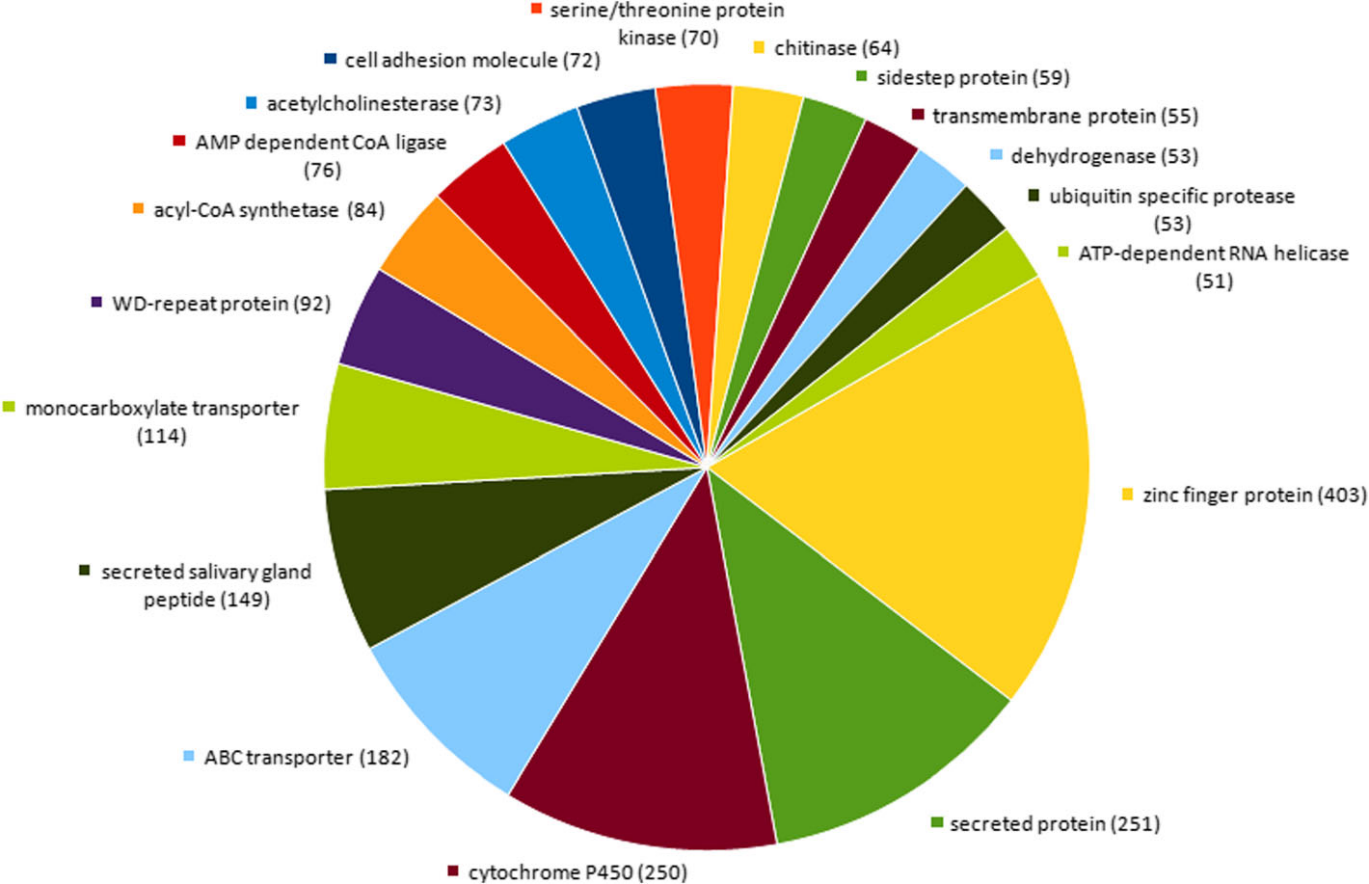
Most prevalent protein categories annotated in the *Ixodes ricinus* genome. Annotations were based on homology to annotated *I. scapularis* proteins. Annotative categories with at least 50 assigned sequences are shown. The number of assigned sequences is given in brackets. The most abundant hits were hypothetical protein (21% of total) and conserved hypothetical protein (13% of total), which were not included into the graph as they do not contain annotative information. A table detailing all annotations and their frequency is provided as Additional file 3

Genomics scaffolds were blasted against the annotated proteins of the closest related species, *I. scapularis* [22]. A total of 25,263 blast results matched the significance criteria of at least 80% amino acid identity and a maximum e-value of 1.0e^-5^. A detailed table of the results including query, hit description, e-value and identity is shown in Additional file 1: Dataset S1. More than 50% of sequences, matching the significance criteria in the blast search described above, reached 95–100% identity with *I. scapularis* sequences (Additional file 2: Figure S1). The annotations of the matching blast hits were reassigned to the *I. ricinus* scaffolds. The frequency of the annotations was counted and is presented in Additional file 3: Dataset S2. Figure 3 shows the most frequently annotated proteins by categories. Hypothetical and conserved hypothetical proteins were the largest groups of annotated proteins. The largest annotated category was zinc finger proteins, followed by secreted proteins and cytochrome P450 coding proteins. No significant shift in the distribution of the most abundant annotations was observed compared to the previous genome version JXMZ01000000.

## Discussion

By flow cytometry of PI-stained nuclei, we estimated that the haploid genome size of *I. ricinus* was on average 2.65 Gb. There were no significant differences in genome sizes observed between the laboratory ticks and the field ticks. Also, the genomes of *I. ricinus* ticks from different geographic origins in Europe were the same size. The genomes of the male *I. ricinus* ticks were in all cases only 95% of the size of their female counterparts (average male genome: 2.57 Gb; average female genome 2.72 Gb). As *I. ricinus* ticks were shown to have an XY sex determination system, this difference likely reflects the amount of DNA distributed on the X and Y chromosomes [23–25]. Despite a diploid set of the same number of chromosomes [23–25], the genome of *I. ricinus* is 0.5 Gb, or 26%, larger than that of its North American relative *I. scapularis*, which is estimated at 2.1 Gb based both on reassociation kinetics and genome sequencing [26]. Similarly, the *I. ricinus* genome is 0.6 Gb, or 33%, larger than the *I. pacificus* genome size estimated by flow cytometry [27]. This is compatible with previous observations of large differences in genome size between species of arthropods in general and ticks in particular.

The genome of the embryo-derived *I. ricinus* cell line IRE/CTVM19 (3.80 Gb) was about 1.4 times the size of the genome of the *I. ricinus* ticks. Multiplication of repeats or insertion of transposable elements across the genome may be more efficient in a rapidly replicating cell in culture than during natural evolution, as has been shown e.g. for *Drosophila* cell lines [28]. Karyotyping of the cell line at passage 179, 6 years prior to the present study, revealed almost similar proportions of cells with 28 (18%),42 (14%), and 56 (16%) chromosomes, corresponding to diploid, triploid and tetraploid sets; the remaining cells were aneuploid (data not shown). By flow cytometry we did not observe this heterogeneity in genome size and our genome size estimate of 3.80 Gb by flow cytometry corresponds best to a predominantly triploid set of chromosomes. Thus, 67 additional passages seem to have favored the selection of cells in the present study with a predominant genome size of 3.80 Gb corresponding either to a triploid set of chromosomes or to the amplification of coding or non-coding regions.

Scaffolding and gap filling is an essential step towards the generation of a high-quality draft genome assembly. New technologies, such as SMRT sequencing, allow the scaffolding of large genomes even without creation of bacterial or yeast artificial chromosomes. To the best of our knowledge (at least at the time of manuscript submission), this is the first application of third generation sequencing, gap filling and scaffolding algorithms in chelicerates. The limitation of high fragmentation due to the shotgun approach with short reads in the published reference genome was overcome by their integration with ultra-long PacBio reads albeit of an inherent lower quality. Thus, the shorter high quality contigs were assembled into long high quality scaffolds: 235,953 contigs were integrated into 204,904 scaffolds. At the same time, the total length spanned was extended by more than 30% from 392,924,918 bp to 515,788,051 bp. The elongation of the assembly is based on the general extension of a high number of contigs rather than an excessive extension of a few contigs. This is reflected by a marked increase of the N50 value by 87% from a contig N50 of 1643 bp to a scaffold N50 of 3067 bp. Due to the combination of the scaffolding with the gap filling approach, only 0.007% of scaffolds contain gaps of more than 25 unknown nucleotides, the longest gap being only 141 bp. By increasing the N50 by 87% and reducing the number of scaffolds containing gaps with unknown nucleotides to 0.007%, we obtained a framework of high quality sequences, facilitating blast searches and alignment approaches against the generated scaffolds. Assuming a similar genome composition for the two closely related species *I. scapularis* and *I. ricinus*, only 30% of the genome consists of coding or unique sequences. Thus, our scaffolds potentially cover 67% of the coding sequences. Estimations of the genome assembly completeness by BUSCO retrieved 55.5% completeness for unique orthologs. Thus, the ratio of unique-repetitive sequences might slightly differ between *I. scapularis* and *I. ricinus* and/or there may be genes unique to *I. ricinus* which do not match any orthology comparison. Because of the high error rate of SMRT sequencing, a very high coverage is required for *de novo* assembly of repetitive regions. This is beyond the scope of this study, which focuses on the coding regions only.

As a first application of the improved genome information, these *I. ricinus* scaffolds of high sequencing quality were blast-searched against proteins annotated in *I. scapularis* in order to match annotations to the *I. ricinus* sequences. In total, 25,263 hits matched the significance criteria of at least 80% identity and a maximum e value of 1.0e^-5^. More than 50% of *I. ricinus* sequences complying with the significance criteria displayed 95–100% identity with *I. scapularis* protein sequences, with similar identity distributions in the different protein categories. This may demonstrate a high level of protein coding sequences conserved between the two species. Besides hypothetical or conserved hypothetical proteins, the most abundant groups comprised zinc finger proteins, secreted proteins and cytochrome P450 coding proteins. Proteins containing zinc fingers are a large and functionally diverse family of structural proteins. The majority of the tick proteins in this category were predicted to bind DNA, RNA or protein and to be involved in gene transcription, DNA replication, translational regulation, protein folding etc. [29]. The secretion of proteins during feeding and digestion is critical for the parasitic lifestyle of the tick. Salivary proteins and secreted salivary peptides, which are also among the largest annotated categories, inhibit the host’s immune system and thus facilitate prolonged feeding of the tick. Cytochrome P450 proteins play, among others, an important role in metabolic resistance, i.e. detoxification of acaricides [30].

## Conclusions

After the publication of the first reference genome of the tick *I. ricinus* [21], the most important pathogen vector in Europe, we have now further extended and annotated the genome sequencing information by third generation sequencing. We propose a first hybrid PacBio-Illumina sequence assembly by scaffolding of the contigs. The assembly was extended by more than 30% and the utility of the scaffolds for genetic research was highly improved by almost doubling the N50 value. We estimated the *I. ricinus* genome size to be 2.65 Gb for laboratory-reared and field ticks from different European countries. The *I. ricinus* genome is 0.5 Gb or 26% larger than the genome of *I. scapularis*, the corresponding North American pathogen vector. Among the 25,000 sequences that we annotated, (conserved) hypothetical proteins, zinc finger proteins, secreted proteins and P450 coding proteins were among the largest protein categories. These categories correlate with important functions of the tick’s lifestyle like e.g. secretion. The annotated sequences pave the way to investigation of the different development stages of the most important European pathogen vector.

## Methods

### Genome size determination by flow cytometry

#### Labeling and fixation of cells

Primary chicken red blood cells (CRBCs; Innovative Research Inc., Novi, MI, USA), cells from the human tetraploid T cell line 1301 (Sigma-Aldrich, St. Louis, MO, USA) and cells from the embryo-derived *I. ricinus* cell line IRE/CTVM19 [31] at passage 246, maintained as described previously [32], were pelleted by centrifugation at 300× *g* for 3 min and washed twice in ice cold Staining Buffer [phosphate buffered saline (PBS; Lonza Walkersville Inc., Walkersville, MD, USA)] containing 2% fetal bovine serum (FBS; Thermo Fisher Scientific, Waltham, MA, USA) and 0.1% NaN_3_ (Sigma-Aldrich).

Adult male and female *I. ricinus* ticks were collected from the vegetation in Luxembourg by the cloth flagging method. Adult male and female *I. ricinus* ticks from laboratory strains from Ireland (Charles River Laboratories, Elphinstone, UK) and Germany (Insect Services, Berlin, Germany) were purchased. The nuclei preparation was adapted from Geraci et al. [27]. Three ticks per gender/origin/biological replicate were snapfrozen in liquid nitrogen, placed in ice-cold PBS containing 50% FBS and ground twice in a 2 cm^3^ dounce homogenizer on ice. The suspension was filtered through a 40 μm cell strainer (VWR International, Radnor, PA, USA) and centrifuged at 80× *g* for 3 min at 4 °C to remove particulate matter. Supernatant was collected and centrifuged at 300× *g* for 3 min at 4 °C. The extracted nuclei were washed twice in ice cold staining buffer and counted in a Neubauer counting chamber.

CRBCs were labeled with the CFSE Cell Division Tracker Kit (BioLegend, San Diego, CA, USA), according to the manufacturer’s protocol. Then, 1 × 10^6^ nuclei from tick samples or cells of the IRE/CTVM19 cell line, CRBCs and the 1301 cell line were each resuspended in 100 μl ice cold Staining Buffer. Five microliters of BV421 Mouse Anti-Human CD45 antibody (Becton, Dickinson and Company (BD), Franklin Lakes, NJ, USA) were added and the nuclei/cells were incubated on ice for 20 min, protected from light. The nuclei/cells were washed twice in 1 ml Staining Buffer. Gentle ethanol fixation was done adapted from Telford et al. [33] as follows. The pellets were resuspended in 0.3 ml PBS with 50% FBS. While gently mixing, 0.9 ml ice cold 70% ethanol (VWR) was added dropwise and the nuclei/cells were incubated overnight at 4 °C.

The fixed nuclei/cells were pelleted at 1000× *g* for 3 min and washed in ice cold Staining Buffer. The nuclei/cells were counted and 500 μl propidium iodide (PI) staining solution (20 μg propidium iodide (Sigma-Aldrich), 200 μg DNase-free RNase A (Thermo Fisher Scientific) in 1 ml PBS) were added per 1× 10^6^ cells. The nuclei/cells were incubated at room temperature for minimum 30 min.

#### Flow cytometry measurement

Samples were analyzed on an LSRFortessa (Becton, Dickinson and Company), using 405 nm and 488 nm and 561 nm lasers with 50 mW output for the detection of BV421 labeling, CFSE and PI staining, respectively. Photomultiplier settings were kept consistent throughout measurements. The results were further analyzed with the FACSDiva software (Becton, Dickinson and Company). Debris and doublets were removed by PI height over width gating. CRBCs were gated by CFSE staining, tetraploid cells by BV421 labeling. A histogram was generated based on the PI fluorescence of gated events. Mean fluorescence was analyzed by separately gating the respective signals. For each sample, three biological replicates were performed and at least 200 *I. ricinus* nuclei or IRE/CTVM19 cells counted per replicate.

#### Calculation of genome size

The mean genome sizes of multiple individual ticks of both sexes for each laboratory tick population, the collected ticks and the cell line were calculated as fold multiples of the CRBC and 1301 standards. Diploid genome size was calculated based on the following conversion formula from Bennett et al. [34]:

$$ \mathrm{Genome}\ \mathrm{size}\ \left(\mathrm{bp}\right)=\left(0.987\times {10}^9\right)\times \mathrm{D}\mathrm{N}\mathrm{A}\ \mathrm{content}\ \left(\mathrm{pg}\right). $$

A total of 2.5 pg of DNA was used as the DNA weight of a diploid CRBC (2C value) as described previously [35]. The coefficient of variation (CV) was calculated by dividing the standard deviation by the average of the distribution of measurements per group. Results with CV < 5% were considered reliable. Differences in genome size between the tick samples and the cell line were analyzed with unpaired Student’s t test (*df* = 4) with *P* < 0.05 as significance threshold using SigmaPlot (Systat Software Inc., San Jose, CA, USA).

### Sequencing

#### High molecular weight DNA extraction

DNA was extracted from 50 *I. ricinus* males (Charles River Laboratories) with the Genomic-tip 20/G kit (Qiagen, Hilden, Germany) with only minimal adaptations of the manufacturer’s protocol. Ticks were ground in a precooled mortar with liquid nitrogen to a fine powder. 2 ml buffer G2 were added to the sample and incubated for 30 min at 37 °C before addition of 0.1 ml Qiagen Protease. Instead of vortexing, the sample was mixed by inversion and incubated at 50 °C for 2 h with gentle agitation. The sample was then centrifuged for 20 min at 13,500× *g* to remove particulate matter. The supernatant was mixed by inversion (instead of vortexing) and applied to an equilibrated Qiagen Genomic-tip. The number of washing steps with buffer QC was increased to four. Final precipitation of the DNA was done by adding 1.4 ml isopropanol to the eluted DNA and immediate centrifugation at 15,000× *g* for 20 min at 4 °C. The DNA pellet was dissolved overnight on a shaker at 4 °C in 10 mM Tris-Cl buffer, pH 8.5. Finally, DNA of smaller size, accidentally fragmented during processing, was removed by size selection using SPRIselect beads (Beckman Coulter, Brea, CA, USA), according to the manufacturer’s protocol, with a bead-to-sample ratio of 0.5. DNA was quantified using the Quant-iTPicoGreen dsDNA Assay Kit (Thermo Fisher Scientific) following the manufacturer’s instructions and sample quality was checked with aNanoDrop (Thermo Fisher Scientific).

#### PacBio and Illumina sequences

High molecular weight *I. ricinus* DNA was sequenced by single molecule real time (SMRT) sequencing with the PacBio RS system (Pacific Biosciences, Menlo Park, CA, USA) at Yale Center for Genome Analysis (West Haven, CT, USA). A 10 kb library was prepared (including DNA fragmentation, DNA repair, adapter ligation, sequencing primer annealing and polymerase binding), according to the manufacturer’s instructions (Pacific Biosciences). A total of 5,624,511 reads with an average read length of 2105 encompassing 11,843,540,746 nucleotides were retrieved.

DNA sequences retrieved from the same laboratory strain of *I. ricinus* ticks by Illumina HiSeq 2500 sequencing were described recently [21] and are publicly available at the DDBJ/EMBL/GenBank Sequence Read Archive under the accession SRP051465. Assembled contigs are accessible within the Whole Genome Shotgun project under the accession JXMZ01000000.

### Bioinformatic analysis

#### Scaffolding, extension and gap filling

Illumina contigs (DDBJ/EMBL/GenBankWGS project JXMZ01000000) were extended, scaffolded and gaps filled based on the PacBio reads by PBJelly [36]. The procedure follows several steps. First, gaps (stretch of minimum 25 nucleotides) were identified in the contigs. Secondly, the PacBio reads were mapped against the assembled Illumina contigs as a reference using BLASR (Basic Local Alignment and Serial Refinement) [37]. Since this algorithm was designed according to the PacBio error model, no further error correction was required for the PacBio raw reads. As a result of the alignment, reads spanning into gaps were identified. The criteria for including reads into the gap filling process were defined as a minimum of 200 bp aligning to the contig ends and at least 25 bp reaching into the gap. Gaps that were too large to be spanned by a single read were closed by the flank-extension approach. This allowed reads extending beyond the flanking contigs to be assembled with overlapping reads matching within 25 bp of the start of the gap and reaching at least 25 bp further into the gap. As a result of this, many gaps were completely filled, others only partially. Therefore, the reads for each gap including 1 Kb of reference sequence from the flanking contigs were assembled with ALLORA (Pacific Biosciences Menlo Park, CA, USA) which is based on the AMOS suite [38]. In order to verify the accuracy of the assembly, we analyzed whether or not the assembly supported the gap in the same way as the read alignments had initially done, i.e. whether flanking sequences and linking sequences in the consensus sequence corresponded to the read alignment. Besides the consensus sequence, the length of the assembled sequence was analyzed for its compliance with the predicted size of the gap. The procedure was applied three times to reach the maximum number of gaps filled, as well as contigs extended and scaffolded with the existing datasets.

#### Genome annotation

Scaffolds obtained by integrating reference Illumina contigs (DDBJ/EMBL/GenBankWGS project JXMZ01000000) with PacBio ultralong reads were annotated based on their homology with the recently published annotated *I. scapularis* genome [22]. This was the only annotated tick genome available at the time of analysis and was thus used as a reference. *I. ricinus* scaffolds were blasted (tblastx) against annotated *I. scapularis* proteins (VectorBase, release IscaW1.4, February 2016) using the default parameters (Word size 3, Expect 10.0, Matrix BLOSUM62). The top hit sequences were extracted in a list format and filtered by an identity threshold of 80% and an e-value of 1.0e^-5^. The results were sorted and grouped according to their annotative description.

## Additional files

Additional file 1:
**Dataset S1.** Homology comparison between *I. ricinus* and *I. scapularis. I. ricinus* scaffolds were blasted against *I. scapularis* scaffolds and annotations of Blast hits matching significance criteria of a maximum e-value of 1.0e^-5^ and minimum 80% identity were transferred to the *I. ricinus* scaffolds. (XLS 2681 kb)
Additional file 2:
**Figure S1.** Sequence distribution as percent identity between *I. ricinus* and *I. scapularis.I. ricinus* scaffolds were blasted against *I. scapularis* scaffolds. Only sequences passing the threshold of a maximum e-value of 1.0 e^-5^ and minimum 80% identity are included. (PDF 229 kb)
Additional file 3:
**Dataset S2.** Number of *I. ricinus* annotations by protein categories. The frequency of assigned annotations was counted. For each annotation, the number of sequences is given. (XLS 354 kb)

## Abbreviations

CRBCs: Chicken red blood cells
CV: Coefficient of variation
SMRT: Single molecule real time
