# Supplementary figures and images for: Genome scaffolding and annotation for the pathogen vector Ixodes ricinus by ultra-long single molecule sequencing
Source: Parasit Vectors. 2017 Feb 8;10:71. doi: 10.1186/s13071-017-2008-9 (PMC5299676; doi:10.1186/s13071-017-2008-9)

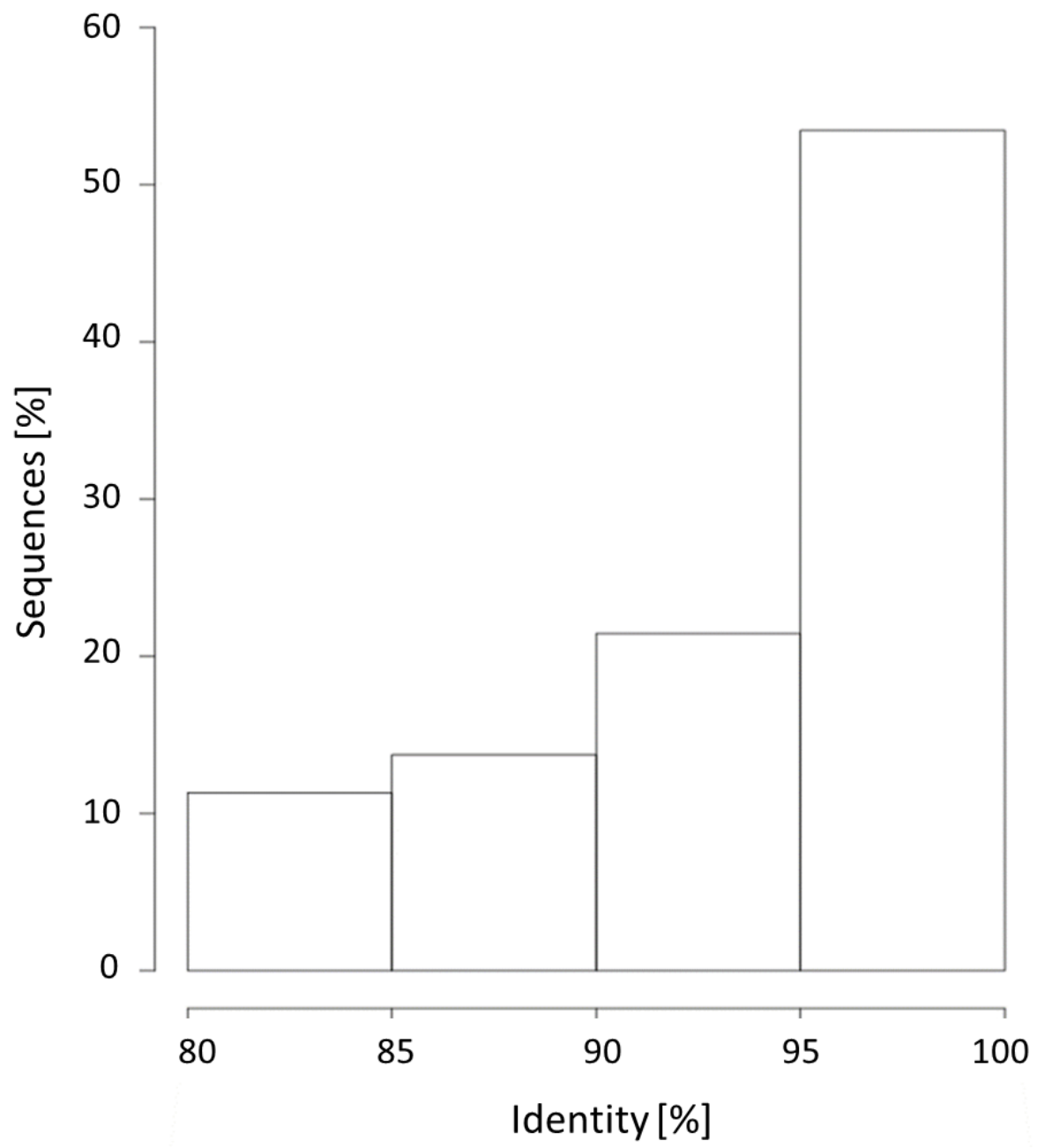

Supplement: Additional file 2: — Figure S1. Sequence distribution as percent identity between I. ricinus and I. scapularis.I. ricinus scaffolds were blasted against I. scapularis scaffolds. Only sequences passing the threshold of a maximum e-value of 1.0 e-5 and minimum 80% identity are included. (PDF 229 kb) [file 13071_2017_2008_MOESM2_ESM.pdf]
